# Role of IL-23 signaling in the progression of premalignant oral lesions to cancer

**DOI:** 10.1371/journal.pone.0196034

**Published:** 2018-04-17

**Authors:** Blaine Caughron, Yi Yang, M. Rita I. Young

**Affiliations:** 1 Research Service, Ralph H. Johnson VA Medical Center, Charleston, SC, United States of America; 2 Department of Microbiology and Immunology, Medical University of South Carolina, Charleston, SC, United States of America; 3 Department of Otolaryngology–Head and Neck Surgery, Medical University of South Carolina, Charleston, SC, United States of America; University of Toronto, CANADA

## Abstract

Mice bearing carcinogen-induced premalignant oral lesions were previously shown to have a pro-inflammatory phenotype, which is replaced with an immune inhibitory phenotype as lesions progress to cancer. Since Th17 cells are prominent at the premalignant lesion state and their levels are supported by IL-23, studies used mice that were IL-23 receptor deficient (IL-23R KO) to determine the requirement for IL-23 signaling in the immunological and clinical status of mice with premalignant oral lesions. The results showed a dependence on IL-23 signaling for the pro-inflammatory state of mice with oral lesions as levels of IL-2, IFN-γ, IL-6, IL-17 and TNF-α were elevated in wildtype mice with premalignant oral lesions, but not in IL-23R KO mice. In contrast, as lesions progressed to cancer, the pro-inflammatory phenotype subsided and was replaced with the inhibitory mediator IL-10 and with Treg cells in wildtype mice, although not in IL-23R KO mice. Clinically, early progression of premalignant oral lesions to cancer was enhanced in IL-23R KO mice compared to progression in wildtype mice. These results show the importance of IL-23 signaling in both the pro-inflammatory phenotype characteristic of premalignant oral lesions and the inhibitory phenotype as lesions progress to cancer.

## Introduction

Tumor development is associated with an infiltration of cells that comprise both host defenses against the tumor and cells that facilitate the tumor’s development. These include tumor-infiltrating T-cells with the potential to react against tumor [1]. However, immune defenses are also subverted by infiltrating macrophages, myeloid-derived suppressor cells and Treg cells [2–4]. There are also instances where the same cell types have both pro-tumorigenic and anti-tumorigenic effects. Tumor-associated macrophages can promote a Th1 anti-tumor effect within premalignant oral lesions [5]. However, together with myeloid-derived suppressor cells, tumor-associated macrophages can also inhibit immune activity in head and neck squamous cell carcinomas, with their presence correlating with upregulated expression of the PD-1/PD-L1 axis [6].

Inflammation has been documented to promote tumor development. For example, inflammatory mediators of Barrett’s esophagus increase the risk of esophageal adenocarcinoma development [7]. The activated immune influx in inflammatory bowel disease has been associated with an increased prevalence of colorectal cancer [8]. Oral leukoplakias, which are associated with an increased development of oral cancer, have increased levels of the inflammatory CXCL12/CXCR4 axis [9]. In oral premalignant leukoplakias, the tumor suppressor GPRC5A (G protein-coupled receptor family C group 5 member A) is repressed, allowing for the inflammatory mediator IL-6 to activate STAT3, which is associated with aggressive oral cancer [10]. A study with oral leukoplakias showed a shift from a pro-inflammatory state to an immune inhibitory phenotype with Treg and inhibitory macrophages during progression to head and neck squamous cell carcinoma (HNSCC) [11]. The conclusion of this study was that chronic inflammation induces immune suppression and tumorigenesis. This shift from an inflammatory to an anti-inflammatory suppressive phenotype with progression from lesions to cancer is consistent with our prior studies showing increased levels of pro-inflammatory cytokines within premalignant oral lesion tissues and a decline within oral cancer [12].

Among the inflammatory cells whose role in cancer development is not resolved are the Th17 cells. Naïve CD4^+^ T-cells can be skewed toward the Th17 phenotype by IL-6 plus TGF-β, and IL-23 is needed to sustain Th17 cells [13, 14]. Th17 cells produce increased levels of the inflammatory mediator IL-17 and most studies suggest a pro-tumorigenic role of Th17 within the cancer environment. Studies with a lung cancer model showed that IL-17 facilitates recruitment of tumor-infiltrating macrophages, increases angiogenesis and enhances tumor metastasis [15]. In studies of colorectal cancer patients, levels of Th17 cells in the blood and lymph nodes were higher in patients with more advanced cancer, and increases in Th17 cells markers were associated with a poorer post-surgical survival [16, 17]. Th17 cells have also been shown to inhibit T-cell anti-tumor immune reactivity in breast cancer and to be associated with poor clinical outcome [18]. In contrast, T-cells of breast cancer patients were shown to have dysfunctional IL-6 signaling responses and, in turn, a reduced capacity to differentiate into Th17 cells; these patients had a higher incidence of post-surgical cancer relapse [19]. IL-17-producing Th17 cells appear to have a dual role in colorectal cancer by triggering the tumor and tumor stroma to produce pro-tumorigenic mediators, while also recruiting anti-tumor protective cytolytic CD8^+^ T-cells [20]. A dual role of Th17 cells is further supported by their capacity to not only produce IL-17, but to concurrently also produce IFN-γ, which is consistent with a Th1-like phenotype, or IL-9, which is consistent with a Th2-like phenotype [21].

In contrast to a likely pro-tumorigenic role of Th17 cells in the cancer environment, less is known about their role in the progression from a pre-cancerous to a cancer state. In a premalignant epidermal squamous lesion model, expression of TGF-β increased expression of Th17-polarizing cytokines, T-cell skewing to a Th17 cell phenotype, levels of CD8 effector cells and IFN-γ-expressing T-cells, and reduced cancer progression [22]. However, neutralizing IL-17 in this same study did not alter the anti-tumor effect of the TGF-β expression, leading the study to suggest that both IL-17-dependent and–independent mechanisms impact on the premalignant environment. Using patient specimens and a carcinogen-induced oral premalignant lesion mouse model, our studies previously showed an increase in Th17 cell levels in premalignant oral lesions, but a decline in these cell levels with an increase in Treg once lesions progress to cancer [12]. To determine if the Th17 cells constitute an attempted response against lesion progression to cancer or facilitate tumor development, our study in the mouse model aimed to sustain Th17 cell levels by treating mice bearing premalignant oral lesions with IL-23 and a TGF-β type 1 receptor inhibitor [23]. The treatment sustained increased Th17 cell levels during lesion progression and sustained increased immune reactivity in the spleen and regional lymph nodes. Clinically, this treatment retarded progression of lesions to cancer. The present study further delved into the role of Th17 cells in the progression from lesions to cancer by assessing the immunological and clinical effects of a deficiency in IL-23 receptor, which would lessen the capacity to sustain Th17 phenotype.

## Materials and methods

### Mice

**Fig 1:**
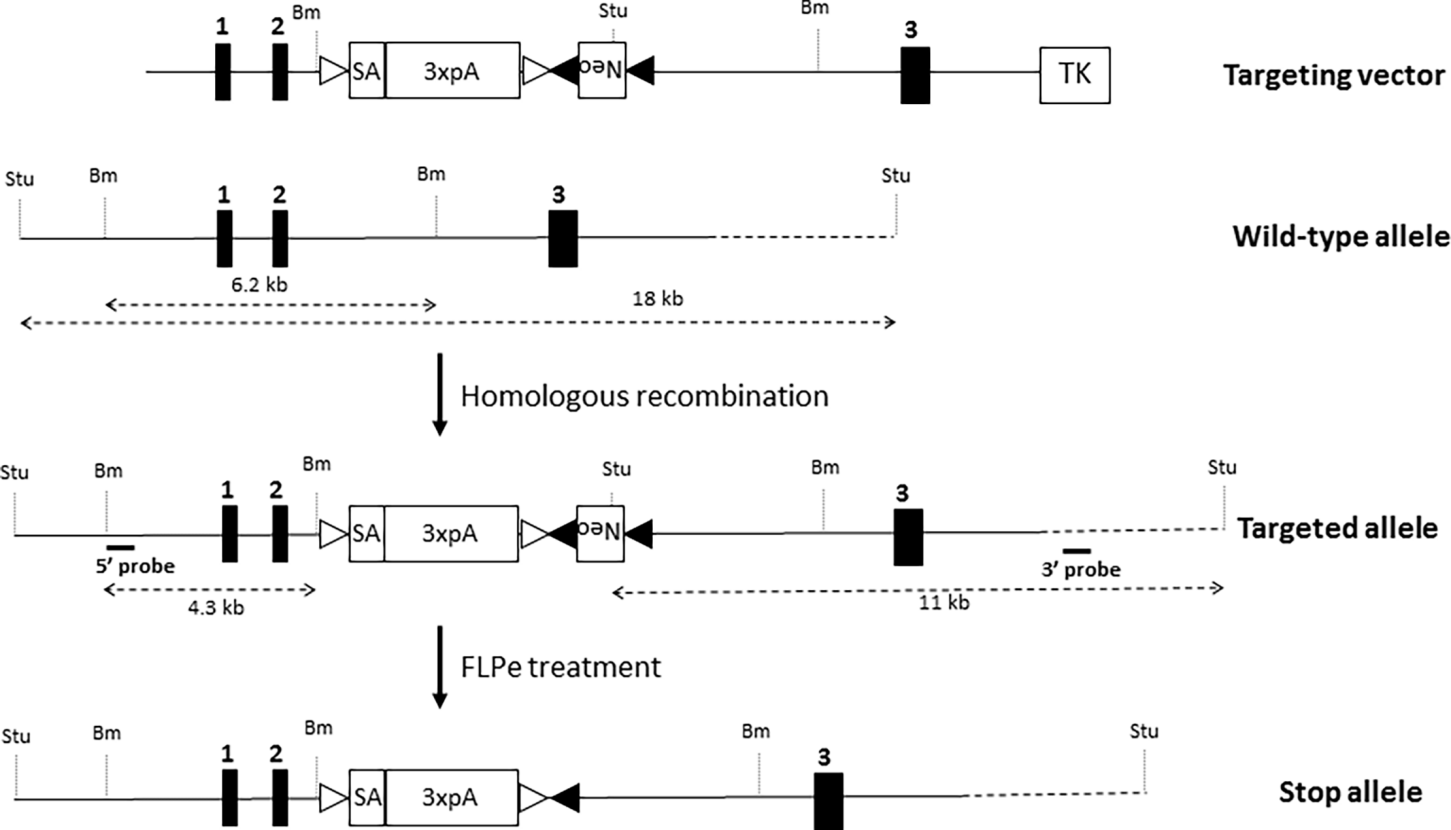
Development of IL-23R conditional knockout mice. Schematic representation of the gene targeting strategy to generate *Il23r* conditional knockout mice. A STOP cassette, consisting of splicing acceptor (SA) and three repeats of poly A signal (3xpA), was inserted into the second intron of the endogenous *Il23r* locus, thus abolishing the expression of IL-23R. The neomycin selection cassette was then removed by crossing with a Flp-expressing mouse line, leaving a single Frt site on the genome. Stu: Stu I restriction site; Bm: BamHI restriction site; open triangle: loxP site; closed triangle: Frt site.

**Fig 2:**
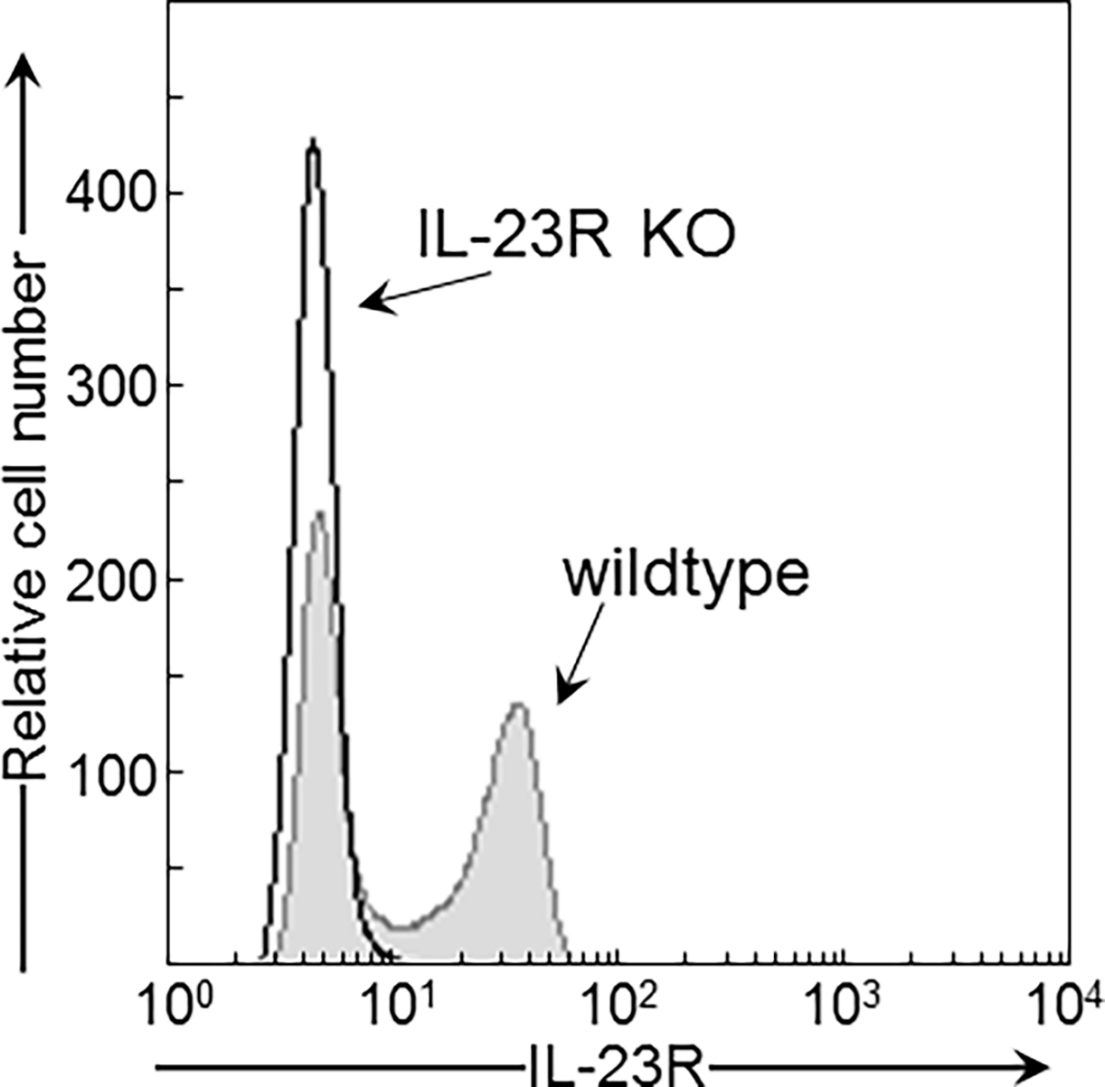
FACS analysis demonstrating the absence of IL-23R expression in the IL-23R KO mice. Spleen cells from wildtype and IL-23R knockout mice were skewed toward activated Th17 cells and then immunostained for IL-23R. Positive-staining cells were identified by flow cytometry.

This study was carried out in accordance with the recommendations in the Guide for the Care and Use of Laboratory Animals of the National Institutes of Health. The protocol was approved by the Institutional Animal Care and Use Committee of the Ralph H. Johnson VA Medical Center of Charleston, SC (ACORP # 582). Wildtype mice used in this study were 2 month old C57BL/6 mice obtained from Charles Rivers Laboratory (Wilmington, MA, USA). IL-23R knockout (IL-23R KO) mice were also used in this study at 2 months of age. A schematic representation of the gene targeting strategy to generate *Il23r* conditional knockout mice is shown in Fig 1. The mouse *Il23r* gene has eleven exons. A STOP cassette, consisting of a splicing acceptor (SA) and three repeats of poly A signal (3xpA), was inserted into the second intron of the endogenous *Il23r* locus. Thus, it abolishes the expression of IL-23R. This stop cassette was flanked by loxp sites, so that it could be removed by the expression of Cre recombinase, e.g. by crossing with transgenic mice expressing tissue-specific Cre. However, the mice that were used in this study were not crossed with Cre mice. Conventional gene targeting was carried out with the E14 cell, an ES cell line on the 129 background. The characteristics of these cells have previously been described [24]. The neomycin selection cassette was then removed by crossing with a Flp-expressing mouse line, leaving a single Frt site on the genome. The IL-23R KO mice were then backcrossed to the C57BL/6 background for ten generations. To confirm a loss of cell surface IL-23R expression, spleen cells of wildtype and IL-23R knockout mice were skewed toward a Th17 phenotype by incubation for 5 days with 10 μg/mL anti-CD3 (Cat. No. MAB484), 5 μg/mL anti-CD28 (Cat. No. AF483), 10 ng/ml mouse TGF-β1 (Cat. No. 100-B), 40 ng/ml mouse IL-6 (Cat. No. 406-Ml), and 10 ng/mL mouse IL-1β (Cat. No. 401-ML) (all reagents from R&D Systems, Minneapolis, MN). They were then immunostained with IL-23R antibody (1:100 dilution; Biolegend, San Diego, CA; Cat. No. 150903) and flow cytometrically analyzed for positive-staining cells (FACSCanto, BD Biosciences, San Jose, CA, USA). This phenotypic analysis showed IL-23R expression on spleen cells of wildtype mice and the absence of IL-23R expression on spleen cells of IL-23R KO mice (Fig 2).

### Treatment to induce premalignant oral lesions

**Fig 3:**
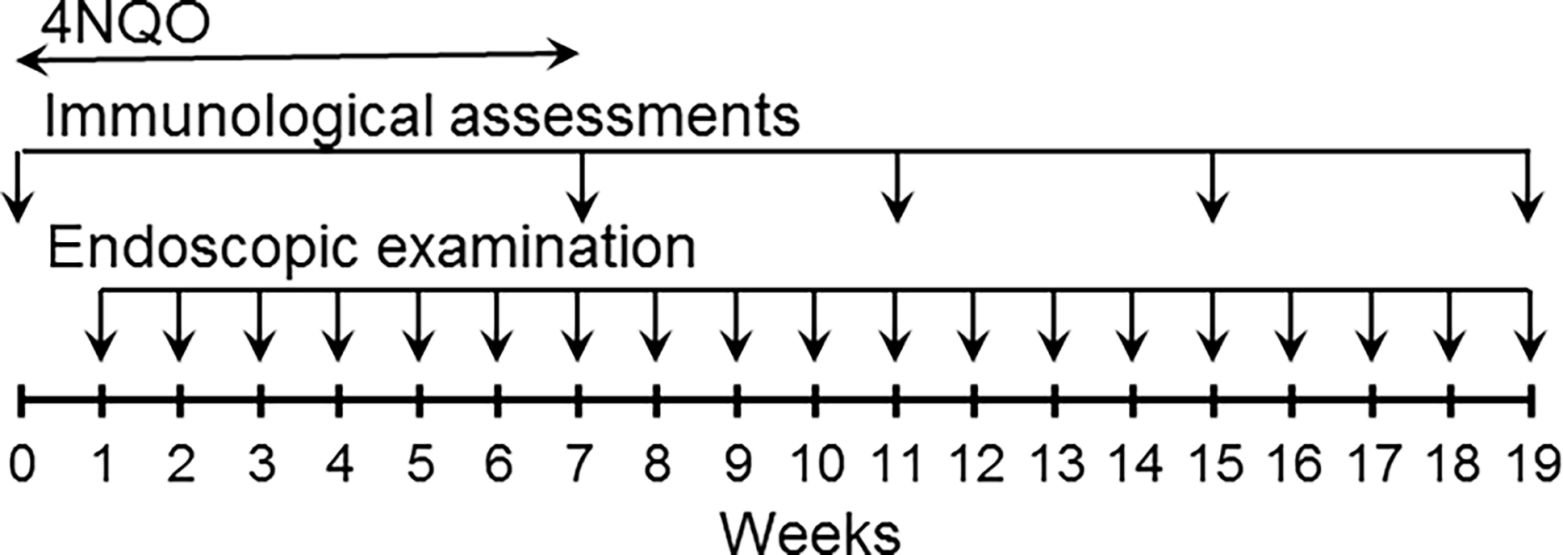
Timelines for 4NQO treatment, endoscopic examination and immunological assessments. Mice were treated with 4NQO in their drinking water for 7 weeks. Once treatment was initiated, they were endoscopically examined at weekly intervals. Subgroups of mice were euthanized for immunological analyses once lesions appeared and at 4 week intervals thereafter.

Starting from 2 months of age, mice were administered drinking water containing 50 μg/ml 4-nitroquinoline 1-oxide (4NQO; Sigma-Aldrich, St. Louis, MO, USA) [25]. Premalignant oral lesions developed on the tongue and their appearance was monitored endoscopically using a 1.9mm x 30° endoscope and a 1088 HD camera (Stryker, Kalamazoo, MI, USA). Once lesions developed at approximately 6–7 weeks of 4NQO treatment, the 4NQO treatment was terminated (Fig 3). Progression of premalignant oral lesions was monitored in groups of 20 mice each on a weekly basis by sedating mice with inhaled isoflurane (Piramal Healthcare, Bethlehem, PA, USA) and examining the oral cavities by endoscopy. Endoscopic images were used for quantitating lesion severity in a blinded manner. Severity was scored between 1 and 4 based on the number of visible lesions, the area of the lesions relative to the area of the tongue, ulceration, thickness, and gross pathology [26]. The parameters for scoring are outlines in Table 1. Scores of 1 to 4 were given for each of the 4 categories, added, and then divided by 4. In prior studies, this scoring method of the overall appearance of the lesions coincided with histologic analysis of paraffin-embedded sections of tongue tissue by an oral pathologist [27].

**Table 1:** Clinical scoring of premalignant oral lesions.

Score	% of tongue surface area	Elevation	Texture	Overall lesion(s)
1	<10%	flat	smooth, soft	single small, homogeneous
2	11–25%	slightly raised papule	wrinkled	single larger, homogeneous
3	26–50%	raised plaque	leathery, surface irregularities	multiple small or single large
4	>50%	grossly exophytic, surface projections	thick, surface irregularities	multiple large, multifocal

### Phenotypic analysis of spleen cells

Once premalignant oral lesions were endoscopically detectable and at 4 week intervals thereafter, (timeline in Fig 3), subgroups of 5 mice each were euthanized by exposure to CO_2_ from a compressed gas tank followed by thoracic incision. Mice that did not receive 4NQO treatment were used as untreated controls. Spleens were collected and homogenized into single cell suspensions. The spleen of each mouse was analyzed individually. All reagents and antibodies that were used for phenotypic analyses were from BD Biosciences. After lysing the erythrocytes, spleen cells were incubated for 4 hours with 50 ng/ml phorbol 12-myristate 13-acetate, 1 μg/ml ionomycin, and brefeldin A solution. To block non-specific staining, they were incubated with FBS and purified rat anti-mouse CD16/32 antibody (1:500; Cat. No. 553142). They were then immunostained with APC-conjugated rat anti-mouse CD4 (1:100; Cat. No. 553051) and FITC-conjugated rat anti-mouse CD8a (1:200; Cat. No. 553031). For IFN-γ intracellular staining, spleen cells were fixed and permeabilized with Cytofix/Cytoperm and then incubated with PE-conjugated rat anti-IFN-γ antibodies (1:100; Cat. No. 562020). For immunostaining Treg cells, spleen cells were stained with reagents in the Foxp3 Treg phenotyping kit as directed by the manufacturer (Cat. No. 560767). Isotype control antibodies included APC-conjugated rat IgG2a (Cat No. 553932), FITC-conjugated rat IgG2a (Cat. No. 553929), and PE-conjugated rat IgG1 (Cat. No. 554685). All antibodies used for staining were monoclonal antibodies. Positively stained cells were visualized and analyzed by flow cytometry (FACSCanto, BD Biosciences).

### Cytokine secretion by spleen cells

Single cell suspensions of spleen cells were seeded into anti-CD3 antibody-coated plates. After 3 days, culture supernatants were collected and their cytokine content was measured using the mouse Th1/Th2/Th17 cytometric bead array kit as instructed by the manufacturer (BD Biosciences; Cat. No. 560485). Cytokines that were measured included IL-2, IFN-γ, IL-6, IL-17A, TNF-α, IL-4, and IL-10. The levels of each cytokine were measured by flow cytometry and analyzed using FCAP Array software (Soft Flow, Inc., St. Louis Park, MN, USA).

### Statistical analysis

Endoscopic examinations were conducted on 20 mice per group. For immunological analyses, subgroups of 5 mice each were euthanized at 4-week intervals, starting from the time that oral lesions were endoscopically detectable. Studies were conducted in triplicate experiments. The results were reported as the mean ± standard error of the mean. They were statistically analyzed for significance by the rank order Mann-Whitney U test. A 95% confidence interval was reported as significant.

## Results

### The absence of IL-23 signaling facilitates the progression of premalignant oral lesions to cancer

**Fig 4:**
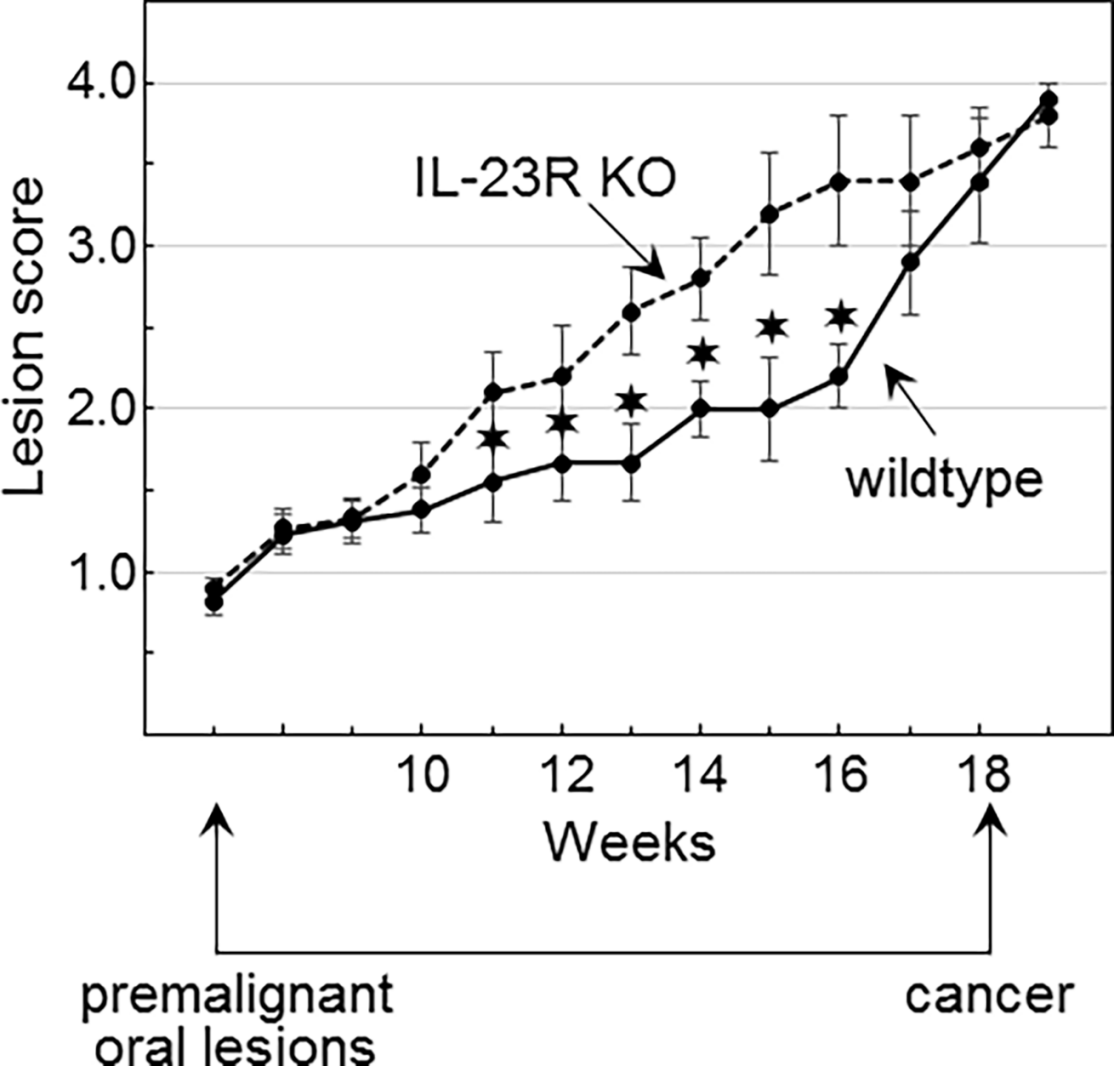
The absence of IL-23 signaling accelerates progression of premalignant oral lesions toward cancer. 4NQO-treated mice were monitored weekly by endoscopy for the appearance of premalignant oral lesions on the tongue. Once lesions appeared, endoscopic images were taken of the tongue and the severity of the lesions was given a score between 1 and 4 by a blinded scorer. Statistically significant differences (*p*<0.05) in the clinical score between wildtype and IL-23R KO mice at each time point is indicated (✶).

**Fig 5:**
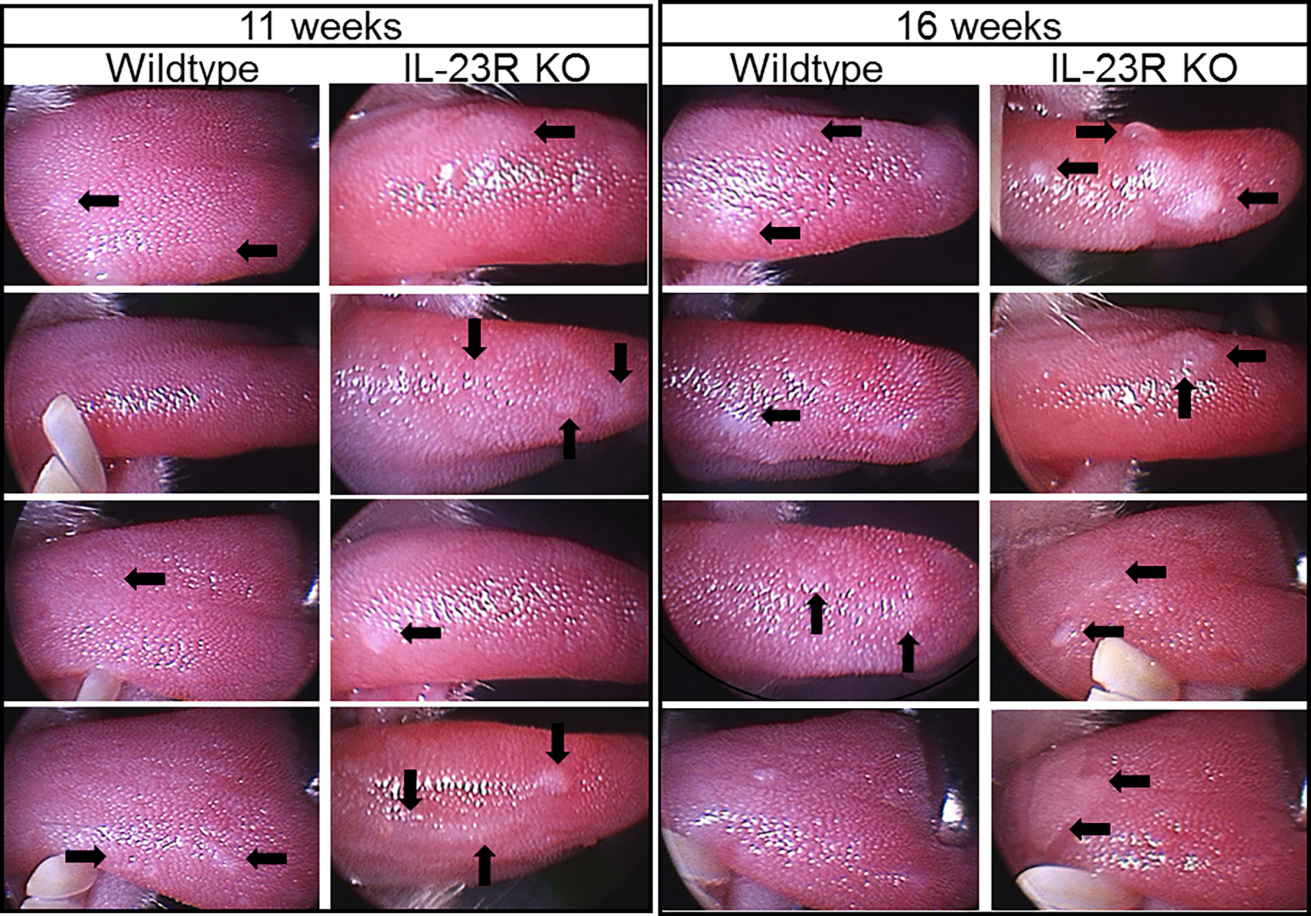
Endoscopic images showing examples of more advanced premalignant oral lesions in IL-23R KO mice. Wildtype and IL-23 KO mice that were treated with 4NQO were monitored endoscopically for the development and progression of premalignant oral lesions on the tongue. Shown are endoscopic images of tongues from 4 representative mice of each group taken at 11 and 16 weeks after onset of 4NQO treatment.

Our prior studies had shown that development of premalignant oral lesions is associated with increased inflammation, which includes increased levels of IL-17 and Th17 cells [12]. Levels of IL-17 and Th17 cells subside as lesions progress closer to cancer. Since IL-23 is needed to sustain Th17 cells, the role of Th17 cells in premalignant lesion development and progression to cancer was determined in 4NQO-treated wildtype mice and IL-23R KO mice. Premalignant oral leukoplakias developed on the tongue, but were not detectable at other sites in the oral cavity. The tongue tissues were objectively graded by the overall gross pathology of the lesions as indicated above in the description of methods and in Table 1. The times at which the lesions appeared on the tongues of wildtype and IL-23R KO mice were similar (Fig 4). However, the lesions that developed on the tongues of IL-23R KO mice progressed more rapidly to a more advanced stage than in the wildtype mice (examples in Fig 5). At 11 weeks, the wildtype mice had lesions that were scored either 1 or 2 in severity, while the IL-23R KO mice had lesions that scored 2 in severity (Fig 5). At 16 weeks, most of the wildtype mice had lesions that scored 2, while the IL-23R KO mice had lesions that scored either 3 or 4 in severity. These results suggest IL-23 signaling limits the rate of premalignant oral lesion progression to cancer. However, at later stages, progression to cancer in the wildtype and IL-23KO mice followed similar kinetics and, thus, appears to be independent of IL-23 signaling.

### Blockade of IL-23 signaling skews immune cell cytokine production in mice with premalignant oral lesions

**Fig 6:**
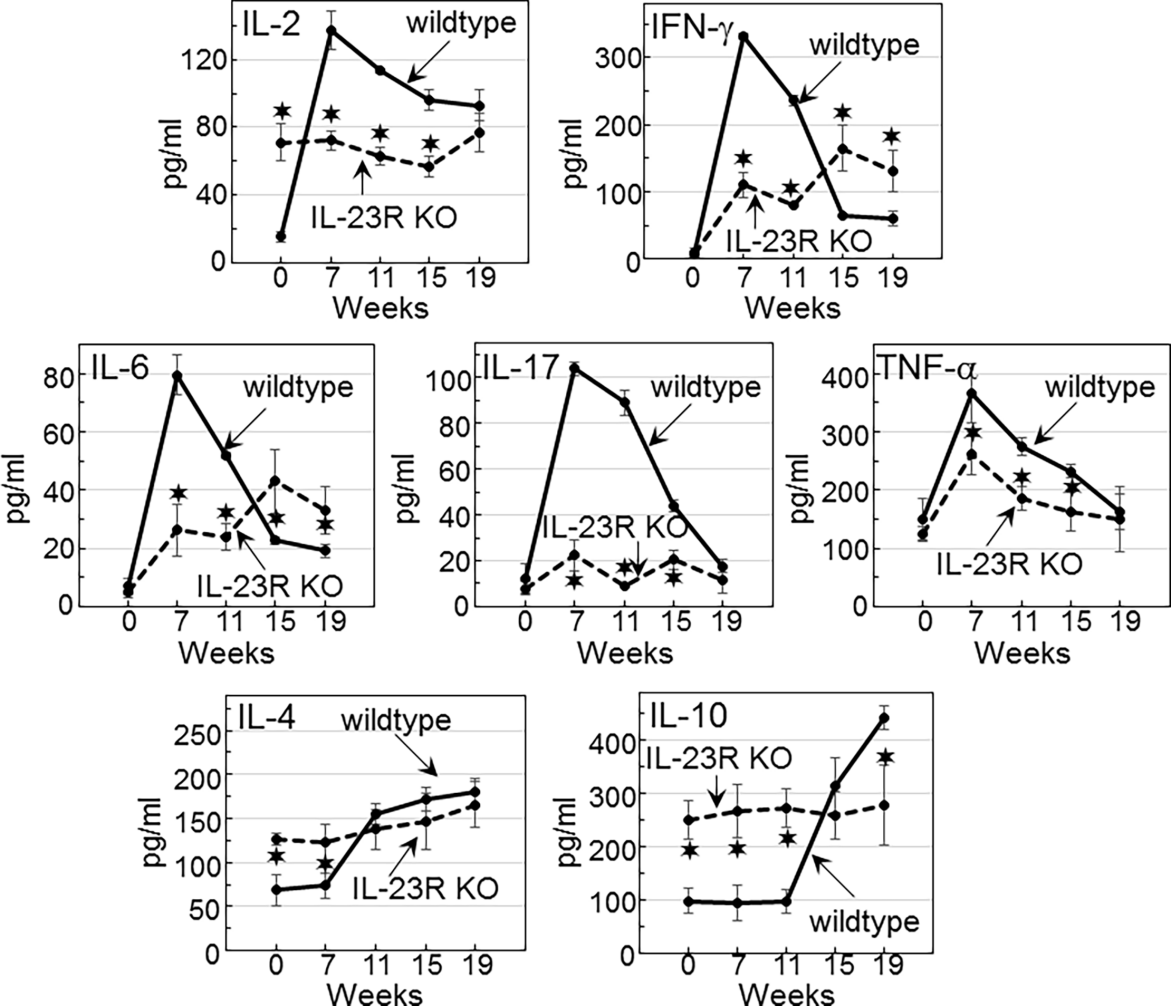
Dependence on IL-23 signaling for the increased spleen cell production of pro-inflammatory cytokines and reduced production of inhibitory cytokines during early stages of premalignant lesion development. Spleen cells of untreated- or 4NQO-treated wildtype or IL-23R KO mice were cultured for 3 days on anti-CD3 antibodies. Culture supernatants were then collected for cytokine quantitation. Significant differences (*p*<0.05) between cytokine levels secreted by spleen cells from wildtype versus IL-23R KO mice are indicated (✶).

Since the above studies showed that a deficiency in IL-23 signaling facilitates oral lesion progression, immune analyses were conducted by comparing cytokine production by spleen cells of wildtype and IL-23R KO mice during the course of lesion development and advancement to cancer. Production of pro-inflammatory mediators, including IL-2, IFN-γ, IL-6 TNF-α and IL-17, by spleen cells of wildtype mice was increased during the development of premalignant oral lesions (Fig 6). As lesions advanced toward oral cancer, production of each of these mediators subsided. In contrast to the increased production of pro-inflammatory mediators during the early stages of lesion development, spleen cell production of the Th2 mediator IL-4 and the inhibitory mediator IL-10 remained low. However, as lesions advanced, production of these inhibitory mediators by spleen cells of wildtype mice increased.

In the IL-23R KO mice, there was the expected reduction in spleen cell production of IL-17 as compared to the increased levels in lesion-bearing wildtype mice (Fig 6). However, in addition to the reduced production of IL-17, production of the pro-inflammatory mediators IL-2, IFN-γ and IL-6 by spleen cells of lesion-bearing IL-23R KO mice was also lower than what was produced by spleen cells of wildtype mice. Spleen cells of the IL-23R KO mice also produced less TNF-α than did spleen cells of control mice, but the difference was less prominent than for the other pro-inflammatory mediators. In contrast to the reduced production of pro-inflammatory mediators by spleen cells of IL-23R KO mice during the early premalignant lesion phase, their production of the inhibitory mediators IL-4 and IL-10 was higher than by spleen cells of wildtype mice. Production of IL-4 by spleen cells of IL-23R KO mice increased slightly as lesions advanced to cancer, at which point similar levels were produced by spleen cells of the IL-23R KO and wildtype mice. IL-10 production by spleen cells of IL-23R KO mice remained at a relatively constant level even as lesions advanced to cancer, although at this latter time point IL-10 production by spleen cell of wildtype mice increased. These results suggest the requirement for IL-23 signaling for the inflammatory phenotype and for limiting the production of IL-4 and IL-10 in the earlier stages of premalignant lesion development and progression.

### IL-23 signaling required for increased levels of CD4^+^ cells and their expression of IFN-γ in mice with premalignant oral lesions

**Fig 7:**
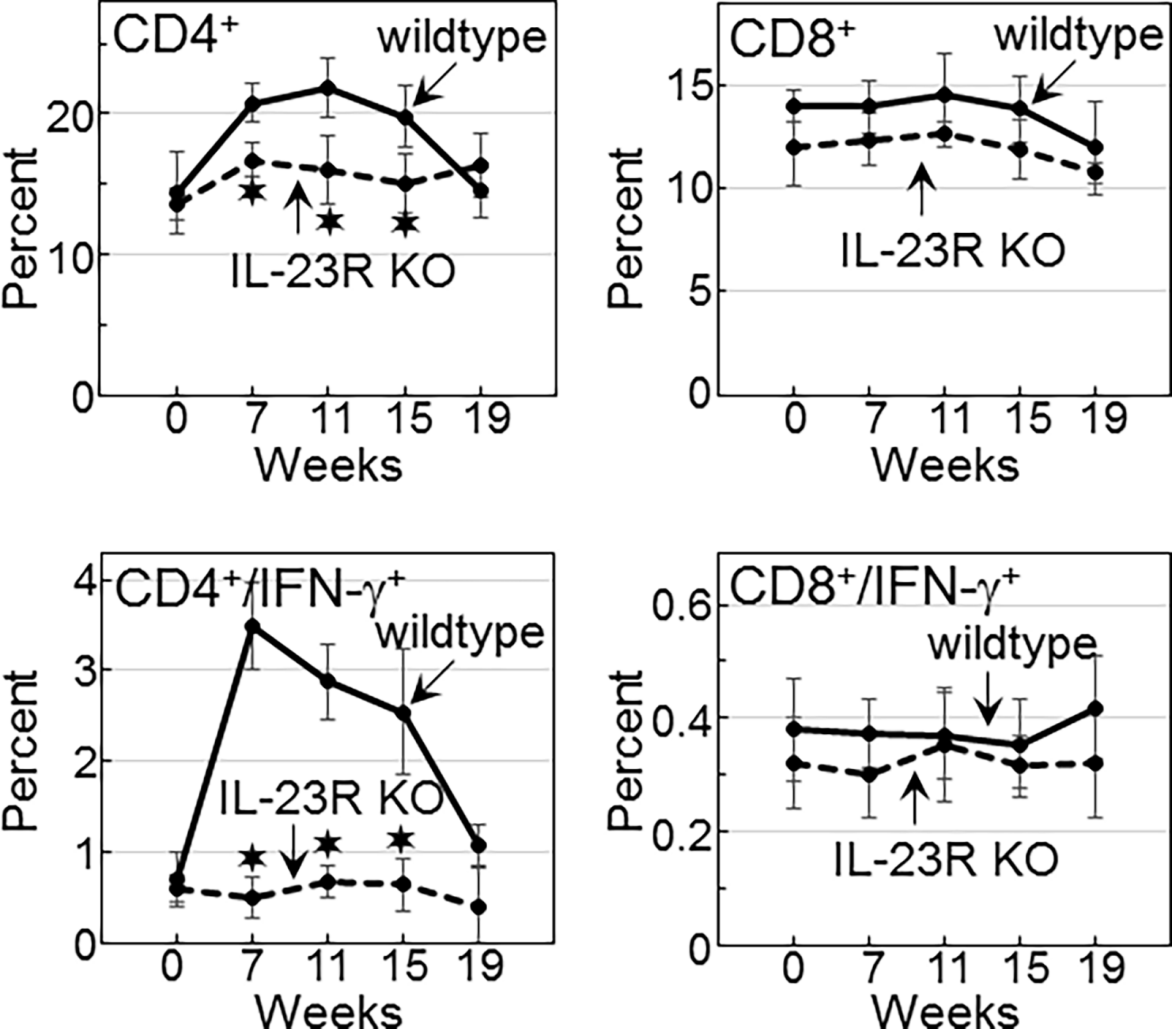
Wildtype, but not IL-23R KO mice, have an increase in splenic levels of CD4^+^ cells and CD4^+^ cells expressing IFN-γ during lesion development, which wanes as lesions advance to cancer. Spleen cells of untreated- and 4NQO-treated wildtype and IL-23R KO mice were immunostained for CD4 and IFN-γ. Cells were analyzed by flow cytometry and significant differences (*p*<0.05) between the percentages of positive-staining spleen cells from wildtype versus IL-23R KO mice are indicated (✶).

**Fig 8:**
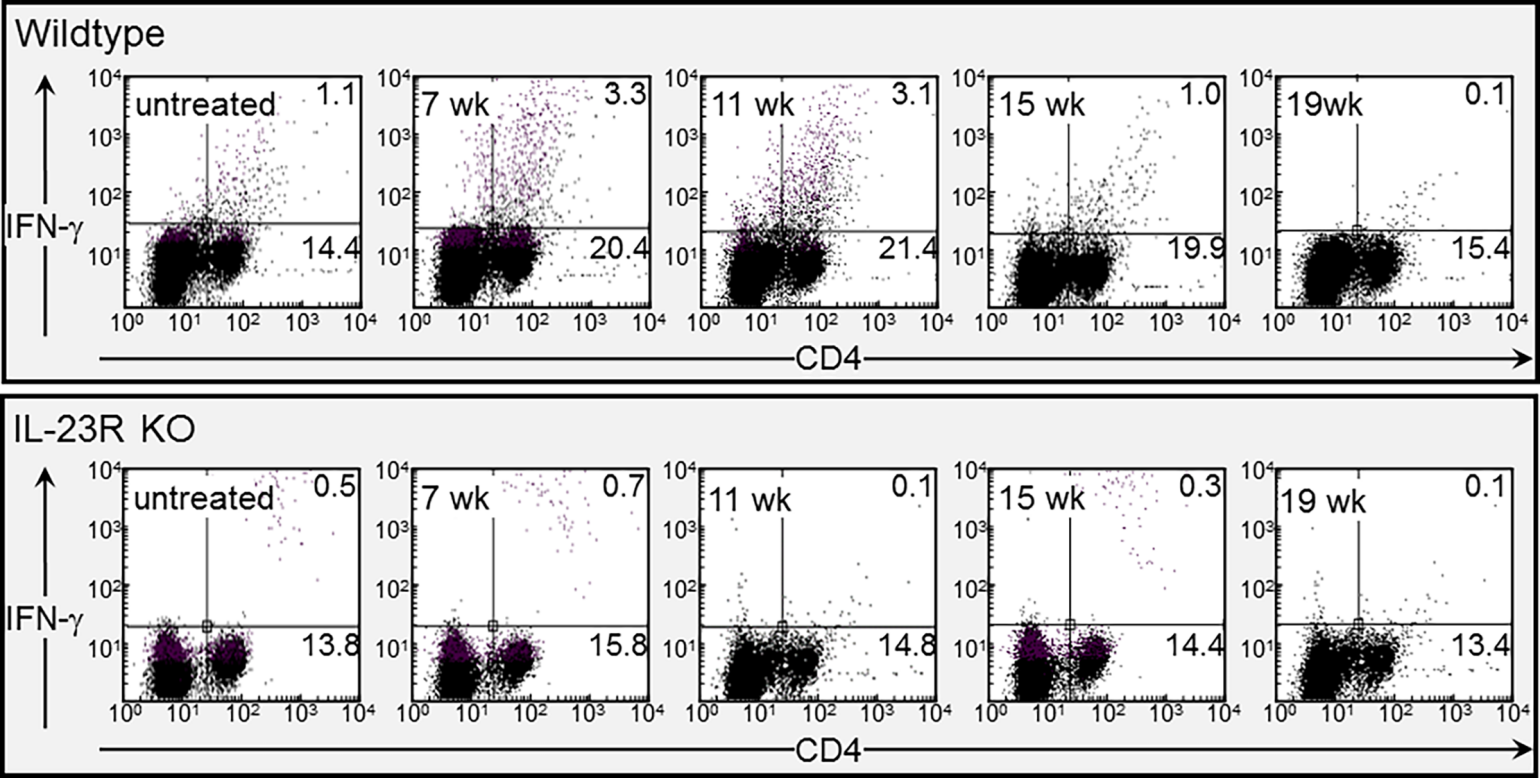
Dot blots showing increased levels of CD4^+^ cells and CD4^+^/IFN-γ^+^ cells in spleens of wildtype mice, but not IL-23R KO mice during lesion development. Spleen cells of untreated- and 4NQO-treated wildtype and IL-23R KO mice were immunostained for CD4 and IFN-γ. Cells were analyzed by flow cytometry. Representative dot blots of analyses of spleen cells from wildtype or IL-23R KO mice are shown.

Because of the differences in cytokine production by spleen cells of lesion-bearing wildtype versus IL-23R KO mice, studies were conducted to determine the impact of blocking IL-23 signaling on the splenic T-cell composition during lesion development and progression to cancer. During lesion development, there was an overall increase the percentage of CD4^+^ cells in the wildtype mice (Fig 7, examples in Fig 8). The increase in CD4^+^ cells did not occur in IL-23R KO mice. As lesions advanced to oral cancer, CD4^+^ cell levels declined in the wildtype mice, while levels in the IL-23R KO mice remained constant.

In contrast to the increase in CD4^+^ cells during the development and progression of premalignant oral lesions in wildtype mice, the percentages of CD8^+^ cells were unchanged (Fig 7). Levels of CD8^+^ cells were slightly lower in the IL-23R KO mice than in wildtype mice, although this difference was not significant. Thus, the development and progression of lesions is associated with an IL-23 signaling-dependent increase in CD4^+^ levels, but not CD8^+^ cells.

Since IFN-γ is crucial for anti-tumor reactivity, phenotypic analyses were conducted to determine how the absence in IL-23 signaling affected CD4^+^ or CD8^+^ cell expression of IFN-γ during the course of lesion development and progression. Consistent with the increase in secretion of IFN-γ by spleen cells of wildtype mice during lesion development, these mice also had an increase in the frequency of splenic CD4^+^ cells expressing IFN-γ (Fig 7, examples in Fig 8). There was no such increase in the IL-23R KO mice during the development of lesions. In the wildtype mice, levels of CD4^+^ cells expressing IFN-γ declined at the later stages during the onset of cancer, while in the IL-23R KO mice, the levels of these cells remained constant throughout the course of lesion development and progression. In contrast to what was seen for CD4^+^ cells, expression of IFN-γ by CD8^+^ cells was unaffected by the development of lesions or their progression to cancer. Levels of CD8^+^ cells expressing IFN-γ were low for both the wildtype and IL-23R KO mice. These results indicate a requirement for IL-23 signaling for the increase in levels of IFN-γ-expressing CD4^+^ cells associated with the development of premalignant oral lesion.

### Increased levels of CD4^+^Foxp3^+^ Treg in wildtype mice bearing advanced lesions but not in lesion-bearing IL-23R KO mice

**Fig 9:**
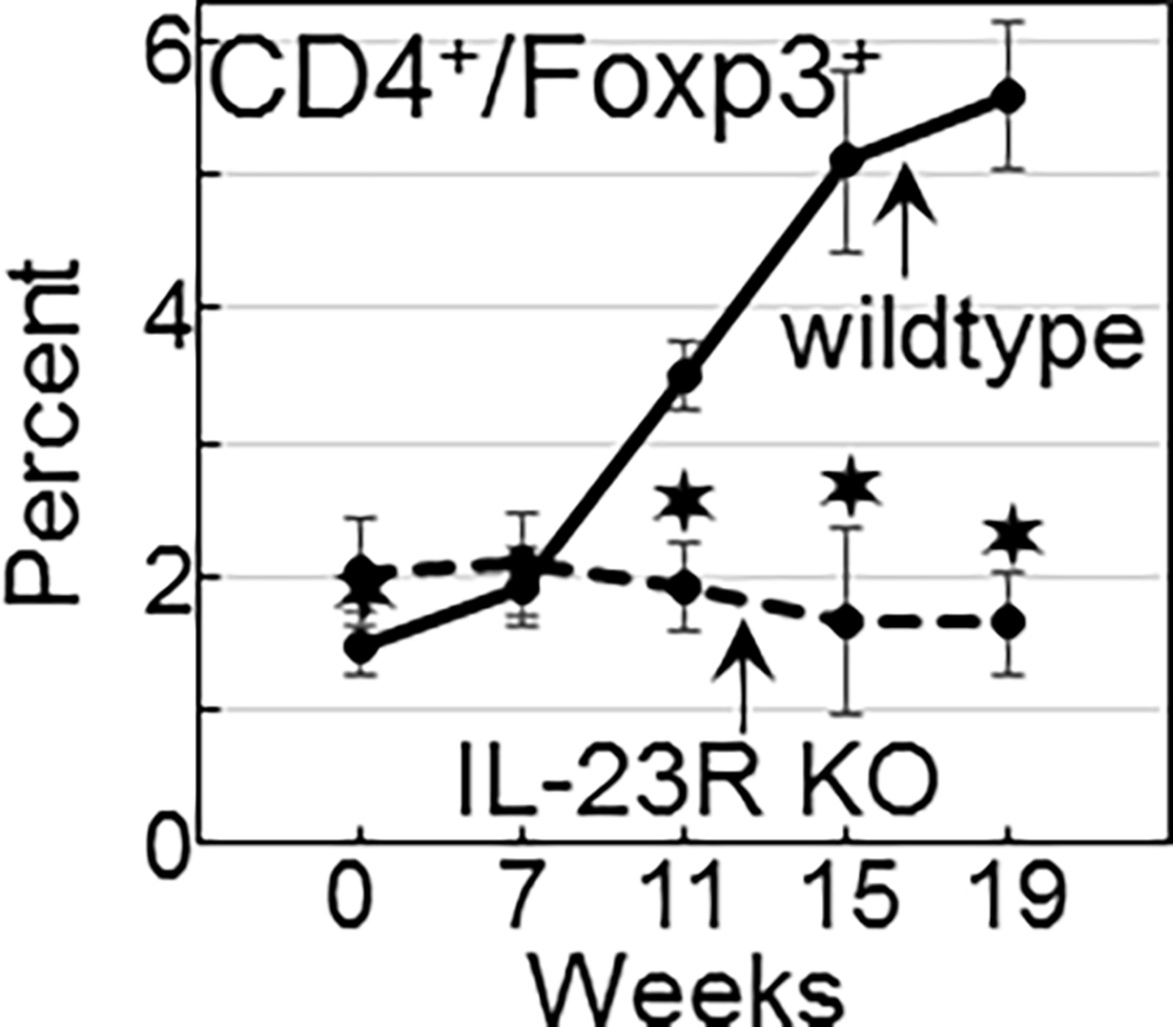
Increased splenic levels of Treg in wildtype mice bearing advanced lesions but not in lesion-bearing IL-23R KO mice. Spleen of untreated- and 4NQO-treated wildtype and IL-23R KO mice were immunostained for CD4^+^ cells expressing Foxp3. Cells were analyzed by flow cytometry and significant differences (*p*<0.05) between the percentages of positive-staining spleen cells from wildtype versus IL-23R KO mice are indicated (✶).

**Fig 10:**
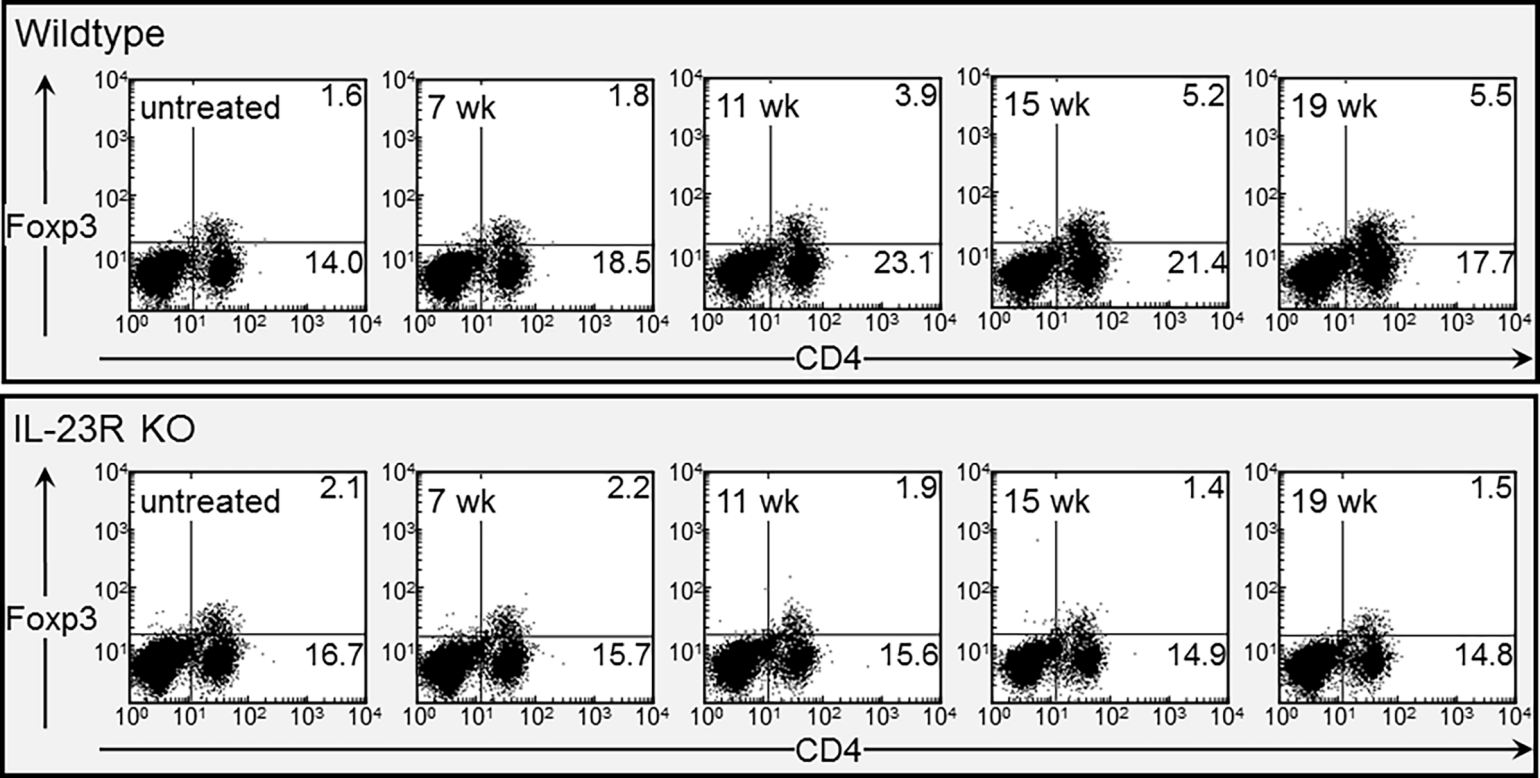
Dot blots showing increased levels of Treg cells in spleens of wildtype mice bearing advance premalignant lesions but not in lesion-bearing IL-23R KO mice. Representative dot blots are shown of spleen cells from untreated- or 4NQO-treated wildtype or IL-23R KO mice that were immunostained for CD4^+^ cells expressing Foxp3.

The above studies showing an IL-23 signaling-dependent decline in spleen cell production of IFN-γ -expressing CD4^+^ cells as lesions progress to cancer with a concurrent increase in spleen cell production of IL-10 prompted studies to determine the levels of Treg in wildtype and IL-23R KO mice. Spleen cell levels of CD4^+^Foxp3^+^ cells in mice with early stage premalignant oral lesions was similarly low for wildtype and IL-23R KO mice (Fig 9, examples in Fig 10). However, as lesion progression advanced, splenic levels of Treg increased in wildtype mice, peaking as lesions developed into cancer. In contrast, levels of Treg remained uniformly low in IL-23R KO mice. These results parallel the changes in the production of IL-10 (Fig 6) by spleen cells during the course of lesion advancement to cancer.

## Discussion

The role of Th17 cells in tumor development and progression is uncertain, with some studies showing their role in promoting tumor development while others demonstrating their presence to reflect attempted reactivity against tumor. For example, Treating Pten-null mice with an inhibitor of Th17 cells or neutralizing IL-17 antibodies reduced their development of prostatic tumors [28]. In contrast, immunological and clinical responsiveness to a vaccine strategy in glioma-bearing mice was associated with increased levels of brain-infiltrating Th17 cells [29]. Since tumor development and progression involves a multitude of steps, it is possible that the role of Th17 cells differs depending on the stage of tumorigenesis. Therefore, the present study examined the inflammatory and inhibitory phenotypes during the course of premalignant lesion development and progression to cancer. The results of these analyses showed increased levels of pro-inflammatory mediators during early stages of lesion appearance and during their progression. However, as lesion progression advanced toward cancer, production of these pro-inflammatory mediators declined and, instead, there was an increase in inhibitory Treg and in production of the inhibitory mediator IL-10.

In an attempt to determine whether the Th17 cells serve a beneficial or detrimental role in the progression of lesions to cancer, we assessed the immunological and clinical effect of moderating the Th17 cell phenotype by using a model that is deficient in IL-23 signaling. These studies showed that, compared to wildtype mice with developing lesions, IL-23R KO mice do not generate the pro-inflammatory phenotype during the course of premalignant lesion development. Contrasting with wildtype lesion-bearing mice, IL-23R KO mice did not produce increased levels of IL-17, IL-2, IFN-γ, IL-6 or TNF-α. The IL-23R KO mice didn’t have increased levels of CD4^+^ T-cells and CD4^+^ cells expressing IFN-γ as is characteristic of wildtype mice with premalignant oral lesions. Furthermore, the IL-23R KO mice lacked the increases in Treg and IL-10 levels that were seen in wildtype mice as lesions advanced to cancer. Levels of CD8^+^ cells and their expression of IFN-γ, while lower than that of CD4^+^ cells, were unaffected by the absence of IL-23 signaling.

The clinical results described in this study showed that premalignant lesions progressed more aggressively toward cancer in the IL-23 KO mice than in wildtype mice. These results supplement an alternate approach we had previously used to assess the role of the Th17 phenotype in limiting progression of lesions to cancer. Instead of limiting the Th17 phenotype as was done in the present study, the prior approach attempted to sustain the Th17 phenotype as lesions progressed by combination treatment with IL-23 and a small molecular weight TGF-β type 1 receptor R inhibitor [23]. The results of that study showed the sustaining the Th17 phenotype was associated with a reduction in lesion progression to cancer. The combination of these studies support the conclusion that the Th17 phenotype is critical in limiting the progression of early premalignant lesions toward malignancy.

It is possible that inflammatory cells such as Th17 cells can have a role in the initial phases of carcinogenesis to promote the transformation process, but then an increase in Th17 cell levels could be an immune attempt to protect against the precancerous lesions. Although the present study assessed the immunological phenotype of mice in the early stages of lesion appearance and as they progressed to cancer, they did not assess whether the inflammatory state appears prior to the detection of lesions. This possibility is, however, supported by studies in a carcinogen-induced colonic inflammation model that showed Th17 cells to be associated with tumorigenesis [30]. This latter study suggested that the inflammatory state could contribute to the cancer development. Our study suggestsTh17 cells are likely to be protective in mice with premalignant oral lesions as lesions advanced toward cancer more readily in the IL-23R KO mice which had a reduced Th17 phenotype. However, once the lesions had advanced, Treg and IL-10 levels increased in the wildtype but not the IL-23R KO mice, suggesting that at the later stages, Th17 cells could be contributing to the cancer–associated immune suppression. This further supports both a beneficial and detrimental role for Th17 cells in the steps that lead to cancer.

While the present study suggests that Th17 cells regulate both inflammatory and inhibitory phenotypes during the course of lesion progression, it is possible that blockage of IL-23 signaling in the IL-23R KO mouse model could have consequences other than limiting Th17 cell presence. For example, in inflammatory disorders, blocking IL-23 also inhibits neutrophil infiltration [31], which can further impact on the inflammatory state of the premalignant lesion environment. While the present study used spleen cells to assess the immunological role of IL-23 signaling in the pro-inflammatory state of mice with premalignant oral lesions, our prior studies have also shown an increased inflammatory state within premalignant oral lesion tissues [26]. We previously showed increased production of IL-23 by primary cultures of premalignant lesion cells compared to IL-23 levels produced by primary cultures from lesions that have progressed to oral cancer [12]. These studies also showed that mediators produced by premalignant lesion cells can stimulate spleen cell skewing toward a Th17 phenotype, which could be a mechanism that contributes to the pro-inflammatory state demonstrated in the present study.

Only a few studies have examined the immunological milieu of premalignant oral lesions. In one such study, the T-cell content of intraepidermal carcinomas was shown to be higher than in SCC tissue [32]. A different study showed that premalignant oral lesions that contain a lower T-cell content have an increased likelihood of progressing to oral cancer [33]. While such studies concluded that the presence of the immune infiltrate indicates immune reactivity against the lesions, additional studies are needed to determine whether immune reactivity is beneficial or detrimental. The combination of our present study that limited Th17 cell levels, and our prior study that sustained Th17 cell levels during the course of premalignant lesion progression [23] showed that the pro-inflammatory phenotype that is associated with the Th17 cell presence tempers the progression of premalignant oral lesions to cancer. Not yet determined is the full mechanisms by which this tempering occurs. The results of the present study suggest that there may be differing regulatory networks associated with IL-23 signaling during early-stage lesions versus during the later state of cancer development. This is based on results showing the dependence on IL-23 signaling to stimulate a cascade the inflammatory cytokines during the earlier stages of lesion progression and to trigger the inhibitory Treg cells and IL-10 during the later stages of progression. Additional studies are needed to gain a better understanding of the role of IL-23 signaling and the pro-inflammatory phenotype associated with premalignant oral lesions in the various stages that lead to cancer development.
